# High-efficiency flexible organic solar modules based on a flexible electrode of a metal grid modified with low-temperature sputtered ITO

**DOI:** 10.1093/nsr/nwag005

**Published:** 2026-01-14

**Authors:** Yongfang Li

**Affiliations:** Beijing National Laboratory for Molecular Sciences, Institute of Chemistry, Chinese Academy of Sciences, China

Small drones are increasingly utilized across diverse applications ranging from agricultural monitoring and infrastructure inspection to logistics delivery and emergency rescue [1]. To maximize flight endurance, integrating photovoltaic power sources is essential. Unlike rigid silicon-based panels, flexible organic solar cells (OSCs) offer a lightweight, conformable solution ideal for the aerodynamics of unmanned aerial vehicle (UAV) wings. However, a critical bottleneck to their practical deployment is the limited efficiency of large-area OSC modules, primarily constrained by the trade-off between electrical conductivity and optical transparency in flexible transparent conductive electrodes (TCEs). The figure of merit (FoM), commonly used to quantify this trade-off, is defined as the ratio of the direct current conductivity (*σ*_DC_) to the optical conductivity [*σ*_OP_(*λ*)], providing a quantitative measure of the balance between transparency and conductivity in TCEs.

Conventional flexible TCEs, such as metal oxides and metal nanowires, are often limited by intrinsic material brittleness that restricts bending capabilities, as well as a relatively low FoM [2]. Transparent metal grid electrodes offer low sheet resistance and high transmittance, with excellent bending stability, but suffer from excessive roughness [3].

Recently, Prof. Zhixiang Wei and co-workers, from the National Center for Nanoscience and Technology, Chinese Academy of Sciences, developed a high-performance TCE and achieved high-efficiency flexible OSC modules applicable to UAVs [4]. They engineered a silver–copper composite grid modified with low-temperature sputtered indium tin oxide (LITO). This innovative design addresses multiple challenges simultaneously.

The LITO layer provides a smooth surface for subsequent layer deposition. It protects the metal grid from oxidation without requiring high-temperature annealing (Fig. 1a), which typically damages flexible substrates. Remarkably, this composite electrode achieved a sheet resistance of less than 1 Ω/□ while maintaining a transmittance exceeding 90%. This balance enables a flexible substrate capable of supporting device fabrication with a high FoM of 4188 (Fig. 1b).

**Figure 1.**
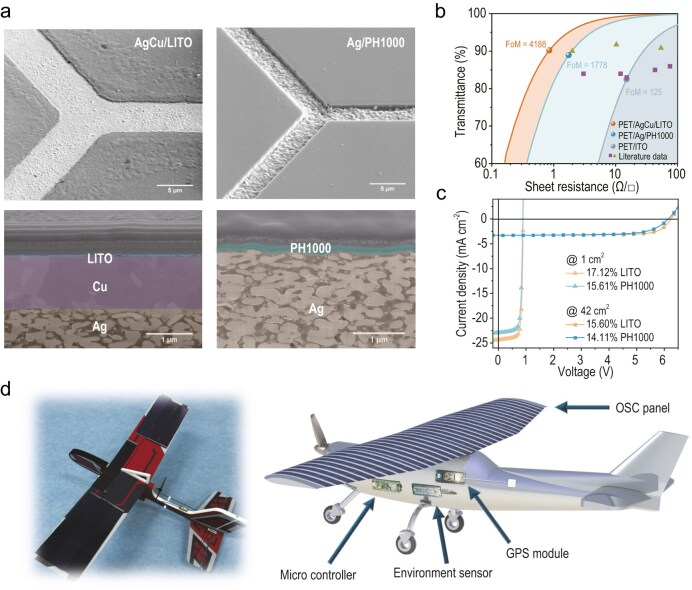
(a) 52° tilt and cross-section scanning electron microscopy (SEM) images of TCEs. (b) FoM comparison of three types of TCEs. (c) Current-voltage (*J–V*) curves of optimized devices based on PH1000 and LITO-modified, respectively. (d) Images of the solar-extended UAV. Adapted from the online file of Tian *et al*. [4].

Using this high-quality TCE electrode, they fabricated flexible *n-i-p* type OSCs with non-halogenated solvent by slot-die coating. The 1 cm^2^ flexible OSCs achieved a record power conversion efficiency (PCE) of 17.12% (Fig. 1c), and 42 cm^2^ modules retained a high PCE of 15.60% (Fig. 1c). Importantly, the unencapsulated devices exhibited high mechanical robustness and environmental stability, retaining 97% of their initial efficiency after 1000 bending cycles (radius = 5 mm) and nearly 90% efficiency after 1080 h of storage under the ISOS-D-1 standard protocol (stored under ambient atmosphere at 25°C and 40% relative humidity) [5].

To demonstrate their practical application, they integrated these flexible solar modules into the wings of a fixed-wing solar-extended UAV (Fig. 1d). The onboard solar-storage system functioned seamlessly, extending the drone’s flight duration by 24.2% and successfully powering sensors for atmospheric data collection.

This work demonstrates important progress on flexible TCEs, and the LITO-modified composite TCE could be widely used in the fabrication of flexible optoelectronic devices.

## References

[1] Floreano D, Wood RJ. Nature 2015; 521: 460–6. 10.1038/nature14542 26017445

[2] Yang F, Huang Y, Li Y et al. npj Flex Electron 2021; 5; 30. 10.1038/s41528-021-00128-6

[3] Qin F, Sun L, Chen H et al. Adv Mater 2021; 33: 2103017. 10.1002/adma.202103017 34369026

[4] Tian C, Zhang H, Zhang Z et al. Natl Sci Rev 2025; 12: nwaf519. 10.1093/nsr/nwaf519

[5] Khenkin MV, Katz EA, Abate A et al. Nat Energy 2020; 5: 35–49. 10.1038/s41560-019-0529-5

